# Elevated beta activity in the nighttime sleep and multiple sleep latency electroencephalograms of chronic insomnia patients

**DOI:** 10.3389/fnins.2022.1045934

**Published:** 2022-11-02

**Authors:** Yuan Shi, Rong Ren, Fei Lei, Ye Zhang, Michael V. Vitiello, Xiangdong Tang

**Affiliations:** ^1^State Key Laboratory of Biotherapy, Department of Respiratory and Critical Care Medicine, Sleep Medicine Center, Mental Health Center, Translational Neuroscience Center, West China Hospital, Sichuan University, Chengdu, China; ^2^Department of Psychiatry and Behavioral Sciences, University of Washington School of Medicine, Seattle, WA, United States

**Keywords:** insomnia, spectral analysis, multiple sleep latency test, polysomnography, electroencephalographic, hyperarousal

## Abstract

**Aim:**

To examine the 24-h hyperarousal hypothesis of insomnia using electroencephalographic (EEG) spectral analysis of overnight polysomnography (PSG) and daytime multiple sleep latency tests (MSLTs).

**Methods:**

Standard PSG and MSLT were recorded in 31 chronic insomniacs (CIs) (21 females, mean age 36.19) and in 21 normal controls (NCs) (18 females, mean age 34.76). EEG spectral analyses were conducted and relative power was obtained for each sleep stage during PSG and each session during MSLTs. Subsequently, CIs were subdivided based on sleep efficiency (SE < or ≥ 85%) or mean sleep latency (MSL) of MSLT (< or ≥ 15 min), and beta power was compared among NCs and CIs subgroups. General liner regression analyses of beta power and PSG parameters were conducted.

**Results:**

CIs had significantly greater beta power in nighttime W, N1, N2, NREM, and in total overnight and in MSLT sessions compared with NCs. CIs with lower PSG-SE or longer MSLT-MSL showed higher beta power at nighttime. Compared with NCs, increased beta power was limited to CIs with lower PSG-SE or longer MSLT-MSL during MSLT sessions. In all subjects, total daytime beta was positively correlated to total overnight beta and MSL, total overnight beta was negatively related to SE. In CIs, total daytime beta and total overnight beta were positively correlated.

**Conclusion:**

Our results support the hypothesis of 24-h cortical hyperarousal in insomnia. We conclude that 24-h cortical hyperarousal is clearly present in insomnia and is greater in insomnia with objective findings.

## Introduction

Insomnia is characterized by prolonged sleep latency, difficulties in maintaining sleep, the experience of poor and non-restorative sleep, accompanied by impaired daytime functioning, including fatigue, exhaustion, and reduced alertness. With marked subjective distress and impairment of daytime functioning, it has been increasingly recognized as a significant public health concern. However, the etiology and pathophysiology of insomnia remain unclear (Morin et al., 2015).

Since 1997 Bonnet and colleagues underscored the importance of hyperarousal concept for insomnia, evidence has accumulated demonstrating that hyperarousal may correlate with disturbance of sleep initiation, maintenance and perception in insomniacs (Bonnet and Arand, 1997; Riemann et al., 2010). Meanwhile, insomnia is often considered as a disorder of hyperarousal at multiple levels, including physiological, cognitive and cortical hyperarousal (Bonnet and Arand, 1997, 2010; Riemann et al., 2010; Zhao et al., 2021). Cortical arousal manifests as increased fast frequency of the sleep Electroencephalogram (EEG) and can be quantitively measured by EEG power spectral analysis (Monroe, 1967; Adam et al., 1986).

Power spectral analysis of EEG has been widely used to explore pathophysiology of insomnia, by assessing the EEG power spectrum and calculating power values in different frequency bands. In healthy adults, high frequency EEG activity predominates in waking state, representing sensory and information processing, and are markers of cortical activation (Perlis et al., 2001b; Kaiser and Lutzenberger, 2005). Low frequency EEG activity predominates in NREM sleep stages. Therefore, increased high frequency EEG power is thought to reflect heightened cortical arousal, while low frequency EEG power is considered an indicator of sleep depth and homeostatic sleep drive. The majority of previous studies of the “hyperarousal hypothesis” focused on EEG spectral power during overnight sleep, and suggested that in insomnia patients compared to controls high frequency EEG activity is increased during NREM sleep but in low frequency activity changes have yielded mixed results (Scientific et al., 1986; Perlis et al., 2001c; Krystal et al., 2002; Buysse et al., 2008; Riemann, 2010; Fernandez-Mendoza et al., 2016; Riedner et al., 2016). Some showed reduced low frequency power in insomnia patients (Merica, 1998; Bastien et al., 2003), whereas others found no differences between insomnia patients and controls (Perlis et al., 2001a,c,; Spiegelhalder et al., 2012). These ambiguous results are likely attributed to the widely varying methodologies employed in these studies (Wu et al., 2013; Kang et al., 2018). Nevertheless, there is considerable evidence supporting that insomnia patients have greater high frequency activity during nighttime sleep providing support for the presence of cortical hyperarousal in insomnia patients (Riemann et al., 2010).

The Multiple Sleep Latency Test, which assesses sleep latency at four or five time points across the day and obtains an average score, the mean sleep latency (MSL), was developed to measure an individual’s level of daytime sleepiness and physiological arousal. Low MSL indicates higher sleep drive and lower arousal level, while high MSL suggests lower sleep drive and higher arousal level. Since the late 80s, extensive data from multiple studies applying MSLT assessment of insomniacs have shown that both shorter sleep time and lower sleep efficiency in a preceding night’s polysomnography (PSG) are associated with longer sleep latency on next-day MSLT (Mendelson et al., 1984; Seidel et al., 1984; Stepanski et al., 1984; Bonnet and Arand, 1998a,b, 2000; Huang et al., 2012). These findings suggested the likelihood that the physiological hyperarousal seen in the nighttime EEG of insomniacs is a round-the-clock phenomenon. However, these two related phenomena, nighttime cortical hyperarousal, and daytime cortical hyperarousal during MSLT as measured by EEG power spectral analysis have not been assessed in the same sample of insomnia patients, which would provide compelling support for insomnia related cortical hyperarousal being a true 24-h phenomenon.

Furthermore, recent studies have identified potential insomnia subtypes. The most biologically severe insomnia phenotypes, determined by objective shorter TST, lower sleep efficiency (SE) or longer MSL, are associated with more clinical risks such as hypertension, type 2 diabetes, mortality, poorer attention-related neuropsychological performance and depression (Vgontzas et al., 2009; Fernandez-Mendoza et al., 2010, 2012; Troxel et al., 2012; Edinger et al., 2013). However, it has yet to be determined whether daytime cortical hyperarousal is associated with different subtypes.

To our knowledge, no study to date has directly examined whether 24-h cortical hyperarousal is present in chronic insomnia and whether cortical hyperarousal varies by insomnia severity subgroups (defined by SE or MSL) *via* quantifying EEG power spectral across both overnight sleep and MSLT of the following day in the same insomnia and control subjects. Furthermore, in previous studies, 24-h hyperarousal was indirectly speculated by elevated beta power in both overnight sleep stages and wake state before sleep which is not equal to daytime wakefulness (Bonnet and Arand, 1997; Riemann et al., 2010; Zhao et al., 2021). EEG data from both overnight PSG and four MSLTs on the following day would be much more representative of the 24-h cortical state. Thus, to better elucidate the neurophysiological mechanisms of chronic insomnia, we aimed to: (1) verify whether chronic insomniacs demonstrate 24-h cortical hyperarousal by comparing EEG power across overnight PSG and daytime MSLT in chronic insomniacs (CIs) with normal control (NCs); (2) explore whether overnight and daytime cortical arousal level vary by insomnia subtype (determined by SE and MSL) by comparing beta power among NCs and insomnia subgroups; and (3) demonstrate whether cortical hyperarousal is a round-the-clock phenomenon by investigating correlational relationships between overnight beta and daytime beta power.

## Materials and methods

### Participants

This was a retrospective study conducted at the Sleep Medicine Center, West China Hospital of Sichuan University. A total of 52 subjects were included in the study, containing 31 chronic insomniacs (CIs) aged 22–62 years old and 21 normal controls (NCs) who are between 21 and 50 years old. The patients were consecutive insomnia patients in our center from January in 2018 to May in 2021.

The CIs fulfilled the following criteria: (1) were diagnosed with insomnia disorder according to Diagnostics and Statistical Manual of Mental Disorder-V (DSM-V) for symptoms (difficulty falling sleep, difficulty maintaining sleep or waking up 30 min before plan and unable to fall asleep again), course of disease (more than 3 times a week and longer than 3 months), severity (causes significant distress or impairment in social, occupational or other important functions) and exclusion (does not attribute to the use of alcohol, medication or other substances, and does not appear exclusively during the course of another mental or medical disorder); (2) aged between 18 and 70 years old; (3) MSL ≥ 8 min measured by standard MSLT; (4) absence of sleep apnea, defined as PSG measured apnea-hypopnea index (AHI) ≥ 5 events/h; (5) absence of sleep related movement disorder, defined as periodic limb movement (PMLI) ≥ 15 based on in-laboratory PSG; (6) absence of sleep onset rapid eye movement period (SOREMP) during both PSG and MSLT; (7) absence of other sleep disorders based on subjective self-reported and objective PSG and MSLT measurements.

The NCs were recruited by posters during the same period, and included college students, the medical and technical staff, and visitors of West China Hospital. All NCs met the following criteria: (1) subjectively reported normal sleep; (2) absence of insomnia related complaints; (3) age between 18 and 70 years old; (4) absence of sleep apnea, as defined above; (5) absence of sleep related movement disorder, as defined above; (6) absence of other sleep disorders based on subjective self-reported and objective PSG and MSLT measurements.

Exclusion criteria for both NCs and CIs included: (1) current use of sedative hypnotics; (2) incomplete PSG or MSLT EEG data; (3) current diagnosis of major mental conditions (i.e., major depression, major anxiety, schizophrenia), severe physical diseases (i.e., acute or chronic heart, hepatic or renal failure); (4) neurological disorder with changed EEG activities (i.e., Parkinson’s disease, Alzheimer’s disease or seizure disorder). Diagnoses of mental or physical disorders were established with clinical history and face-to-face physician interview.

Furthermore, CIs with objective shorter TST, lower SE or longer MSL are associated with daytime physiological hyperarousal and higher risk of some mental and physical disease (Vgontzas et al., 2009, 2012; Fernandez-Mendoza et al., 2010, 2012). Considering without a fixed PSG recording time in current study and correcting differences in sleep opportunities between subjects, SE instead of TST was used to measure objective sleep duration. An SE of 85% or higher is considered normal for healthy adults between 35 and 49 years old (Boulos et al., 2019). Therefore, we subdivided the CIs into insomnia SE ≥ 85% and insomnia SE < 85%. While there is an absence of an agreed-upon comparable criteria for MSL in the literature, the median of MSL (MSL 15 min) in CIs was used as the subgroup criterion, following a published example (Li et al., 2015). This study was approved by the Research Ethics Board of the West China Hospital of Sichuan University, and informed consent was obtained for all subjects.

### Measurements

#### Demographics, clinical characteristics, and physical examination

Prior to overnight PSG recording, face-to-face interview and a series of clinical tests were performed on each subject to collect demographic characteristics and medical history, and to complete physical examination including height, weight, the size of neck, waist and hip, use of cigarettes, alcohol and medications, previous and current diagnosis of major mental and physical disorders.

#### Polysomnography

Each subject underwent an overnight PSG examination in sleep laboratory of sleep medicine center in West China Hospital of Sichuan University including electroencephalogram (EEG), electrooculogram (EOG) and electromyogram (EMG) recordings. Following the American Academy of Sleep Medicine (AASM) Manual for Scoring of Sleep and Associated Events (Berry et al., 2017), PSG recording included six channels (i.e., F4-M1, C4-M1, and O2-M1 as recommended EEG channels; F3-M2, C3-M2, and O1-M2 as alternative channels) and EMGs of submental and anterior tibialis. Respiration was monitored with oral-nasal airflow, rib cage and abdominal movements, and arterial oxygen saturation of index finger. Participants were allowed to follow their habitual sleep schedule and were monitored continuously for 8–10 h. To ensure data quality, a sleep technician continuously monitored the overnight PSG recordings. The sleep records were scored according to AASM criteria by a senior EEG expert (Fei Lei) blinded to subject group. The morning after the PSG subjects completed a brief morning sleep assessment questionnaire.

The sleep and related measures extracted from the scored PSG included: total sleep time (TST), sleep latency (SL), time of wake after sleep onset (WASO), time spent in sleep stages 1–3 (N1–N3), in total non-rapid eye movement sleep (NREM), and in rapid eye movement sleep (REM), sleep efficiency (SE), AHI and PMLI.

#### Multiple sleep latency test

Daytime sleep EEG measures were obtained from Multiple Sleep Latency Test (MSLT) protocols, which were performed the day after the overnight PSG recording. Following the AASM practice parameters for Clinical Use of the MSLT (Littner et al., 2005), consecutive four sessions of MSLT started at 09:00 a.m., and every session provide 20 min nap opportunities with an interval of 2 h. The first 30 s epoch of any sleep stage occurring was determined to be sleep onset, or 20 min was assigned to sleep onset when the subject did not fall asleep within 20 min. The MSL was calculated by averaging the sleep latency scores of the four sessions. To ensure data quality, a sleep technician continuously monitored the daytime MSLT recording. Before the start of each of the MSLT bouts self-reported sleepiness was assessed by Stanford Sleepiness Scale (SSS) (MacLean et al., 1992).

#### Spectral analyses

We focused on spectral analyses on C3 or C4 channel during overnight PSG and MSLT, consistent with previous insomnia research (Perlis et al., 2001c; Krystal et al., 2002; Fernandez-Mendoza et al., 2016; Li et al., 2021). EEG signals were sampled at 500 Hz. EEG data were filtered between 0.5 and 40 Hz *via* Butterworth filter before artifacts exclusion. Visually inspection was applied to exclude artifacts; therefore, EEG epochs (30 s) containing arousal, movements, and various artifacts (including EMG, etc.) during sleep, and 10 s around the translation between sleep stages were excluded. Accepted EEG data were subjected to spectral analyses using a commercial technical software package, written in Python and developed by Synwing Technologies Co., Ltd.^1^ The power spectral density was calculated by applying Welch’s method, with a 5-s Hanning window and 50% overlap. Specifically, a Fast Fourier transform (FFT) algorithm was used to calculate absolute and relative spectral power, with a Hanning window applied before FFT. The FFT analyzed the data as a 5-s small period spectral analysis of a sliding process on 6 consecutive 5-s small periods, to yield a 30-s average value of EEG power per 30 s epoch. Each 30 s epoch corresponded to a sleep or wake stage, so 30 s power values under the same stage were selected and averaged to obtain power values of wake and each sleep stages.

Relative power is more sensitive and can reduce individual differences (i.e., thickness of skull, fat or other soft tissues) relative to absolute power (Zhao et al., 2021). Relative power is computed by dividing the absolute power of each frequency band by the sum of absolute power in all frequency bands. We calculated relative power for frequency bands of delta1 (0.5–1.0 Hz), delta2 (1.0–4.0 Hz), theta (4.0–8.0 Hz), alpha (8.0–13.0 Hz) sigma (13.0–16.0 Hz), beta (16.0–30.0 Hz) (Steriade, 2006; Kurylenko et al., 2015). Delta1 and delta2 activities have different neural generators, therefore, we subdivided delta into two frequency bands (Steriade, 2006).

For overnight PSG, EEG data in wake (2 min artifact-free EEG before the occurrence of the first sleep stage), each sleep stage (NREM1, NREM2, NREM3, and REM), total NREM (all selected NREM sleep stages) and total overnight (including wake and all sleep stages) were used for spectral analyses. Consistent with the PSG EEG data and considering the limited amount of useful MSLT EEG data, MSLT EEG data selection included both wake (2 min artifact-free EEG before the first occurrence of sleep) and all artifact-free sleep stages recorded were combined to calculate power values for each MSLT session. All four sessions of MSLT EEG data were summed into total daytime EEG data for spectral analyses. The power values of MSLT wake before sleep onset and all subsequent artifact-free sleep stages in each MSLT session were also separately calculated.

### Statistical analysis

Prior to statistical testing, the data were examined for normality using Kolmogorov-Smirnov testing. Previous reports indicate that the distribution of both absolute and relative power best approximate a normal distribution with a logarithmic transformation (John et al., 1980; Gasser et al., 1982; Krystal and Edinger, 2010); consequently a lg10 transformation of relative power was utilized in the analyses. An Independent *t*-test or Mann-Whitney *U*-test for normally or non-normally distributed continuous variables, or Chi-square (χ^2^) test for categorical variables was used to examine demographic characteristics, overnight EEG power and total daytime EEG power between NCs and CIs groups. The Kruskal-Wallis H test or Analysis of Variance (ANOVA) was used to evaluate differences of demographic characteristics, overnight EEG power and total daytime EEG power among NCs and SE/MSL determined insomnia subgroups, and Bonferroni correction was used to account for the multiple two-group comparisons. Repeated-measures ANOVA with *post hoc* analysis of within-group and between-group effects examined how EEG power changed across MSLT sessions, between groups, as well as the interaction of sessions and groups.

To evaluate potential statistical power when treating SE and MSL as continuous variables and to evaluate whether cortical hyperarousal is a round-the-clock phenomenon, general linear regression analyses were performed between nighttime beta and daytime beta, nighttime beta, and SE and MSL, and daytime beta and SE and MSL within all subjects, NCs and CIs. Statistical analyses were performed with *SPSS* version 26.0. All statistical tests were 2-sided, and *P*-value and adjusted *P*-value for multiple comparison of less than 0.05 were considered statistically significant.

## Results

### Demographics and polysomnography characteristics of the participants

A total of 52 subjects satisfied inclusion and exclusion criteria and were enrolled for analysis, including 21 NCs and 31 CIs. An Independent *t*-test or Mann-Whitney *U* test or Chi-square (χ^2^) test was used to evaluated demographic characteristics and PSG parameters between NCs and CIs. There were no statistically significant differences between NCs and CIs in age or sex distribution. Compared with NCs, CIs had significantly shorter TST, less NREM, lower SE and longer SOL and MSL. However, WASO, N1, N2, N3, REM, and ESS score were similar between the two groups. Demographic and PSG characteristics of all participants are provided in Table 1.

**TABLE 1:** Demographics and overnight PSG measurements in normal controls and chronic insomniacs.

	Normal controls (*n* = 21)	Chronic insomniacs (*n* = 31)	ν	t/χ^2^/u	*P*
Age (y)^u^	34.76 (8.38)	35.68 (10.69)	–	312.500	0.808
Sex (m/f)^χ2^	3/18	10/21	1	2.157	0.142
MSL (min)^t^	12.20 (3.86)	14.97 (3.42)	51	–2.716	0.009*
ESS^u^	7.24 (4.25)	5.81 (5.08)	–	262.000	0.235
TST (min)^u^	447.85 (43.49)	399.19 (83.87)	–	191.00	0.012*
SE (%)^u^	88.51 (7.86)	78.31 (16.09)	–	177.000	0.006*
SOL (min)^u^	12.52 (13.79)	31.88 (55.42)	–	434.500	0.042*
WASO (min)^u^	45.07 (32.74)	75.61 (57.46)	–	423.500	0.068
NREM (min)^t^	355.01 (30.39)	322.26 (63.93)	45.722	2.470	0.017*
N1 (min)^u^	70.26 (42.37)	61.94 (26.71)	–	305.500	0.709
N2 (min)^t^	251.68 (39.62)	235.02 (68.61)	48.995	1.106	0.274
N3 (min)^u^	33.10 (25.22)	25.10 (25.49)	–	251.500	0.167
REM (min)^t^	90.25 (28.98)	77.05 (34.11)	51	1.453	0.153

PSG, polysomnography; TST, total sleep time; SOL, sleep onset latency; WASO, wake after sleep onset; N1, duration of non-rapid eye movement sleep stage 1; N2, duration of non-rapid eye movement sleep stage 2; N3, duration of non-rapid eye movement sleep stage 3; NREM, time of whole non-rapid eye movement sleep; REM, rapid eye movement sleep time; SE, sleep efficiency; MSL, mean sleep latency of four multiple sleep latency tests; ESS, Epworth Sleepiness Scale.

*P ≤ 0.05; ^t^, comparison between Normal Controls and Chronic Insomniacs by Independent *t*-test; ^u^, comparison between Normal Controls and Chronic Insomniacs by Mann-Whitney *U* test; ^χ2^, comparison between Normal Controls and Chronic Insomniacs by Chi-square (χ^2^) test.

### Comparison of overnight power between normal controls and chronic insomniacs groups

**FIGURE 1:**
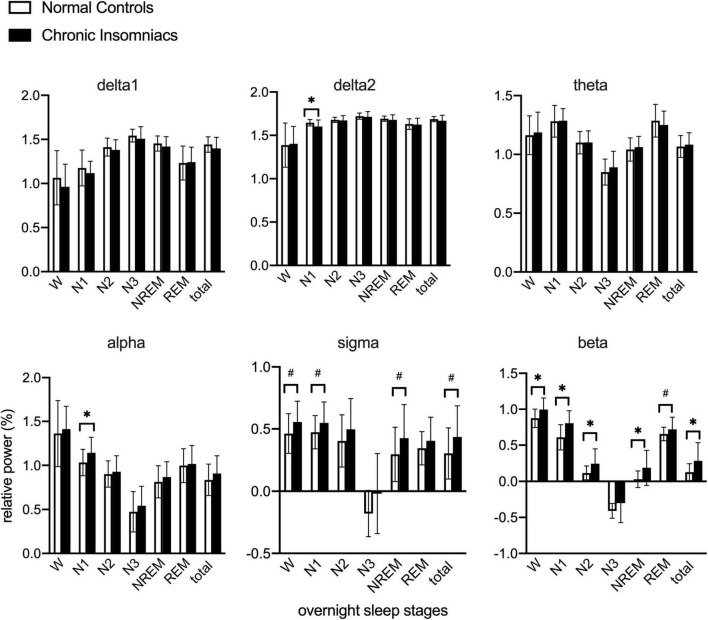
Lg10-transformed relative power of all frequency bands during overnight W and different sleep stages in normal controls and chronic insomniacs. W, 2 min before sleep onset; N1, non-rapid eye movement sleep stage 1; N2, non-rapid eye movement sleep stage 2; N3, non-rapid eye movement sleep stage 3; NREM, non-rapid eye movement sleep; REM, rapid eye movement sleep; Total NREM, containing N1, N2 and N3 sleep stages. Total overnight, including W and all sleep stages. delta1, 0.5–1.0 Hz; delta2, 1.0–4.0 Hz; theta, 4.0–8.0 Hz; alpha, 8.0–13.0 Hz; sigma, 13.0–16.0 Hz; beta, 16.0–30.0 Hz. *P ≤ 0.05; ^#^0.05 < *P* ≤ 0.1.

Based on overnight PSG recording, power in delta1, delta2, theta, alpha, sigma, and beta frequency bands in NCs and CIs are reported in Figure 1. Independent *t*-test was used to examine overnight EEG power between NCs and CIs groups. Compared to NCs, CIs significantly increased beta power in W, N1, N2, NREM, and whole night, increased alpha in N1, and decreased delta2 power in N1. However, no other differences between groups in any frequency band in W, each sleep stage and whole night were noted.

### Comparison of daytime power between normal controls and chronic insomniacs groups

**FIGURE 2:**
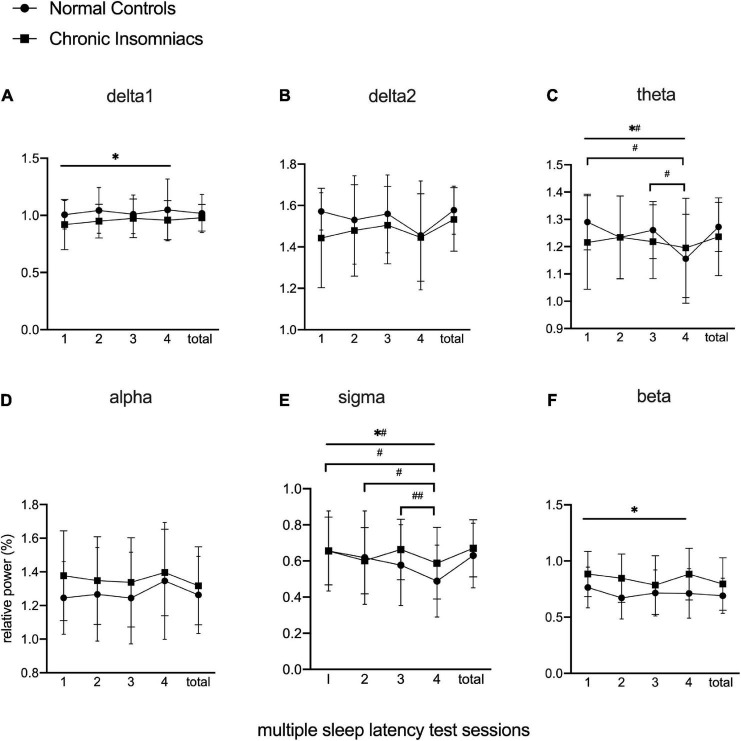
Lg10-transformed relative power of all frequency bands during MSLT in normal controls and chronic insomniacs. Sessions, consecutive four multiple sleep latency tests. Total, the sum of four multiple sleep latency tests in relative power. delta1, 0.5–1.0 Hz; delta2, 1.0–4.0 Hz; theta, 4.0–8.0 Hz; alpha, 8.0–13.0 Hz; sigma, 13.0–16.0 Hz; beta, 16.0–30.0 Hz. *Statistically significant in main group effect; ^*#^statistically significant in group × session interaction effect; ^#^statistically significant in *post hoc* analysis of simple session effect within normal control; ^##^statistically significant in *post hoc* analysis of simple session effect within chronic insomniacs.

Figure 2 depicts effects of session, group, and session × group interaction in each frequency band during daytime MSLT *via* repeated-measures ANOVA with *post hoc* analysis of within-group and between-group effects. Delta1 and beta power showed significant main group effects. In CIs, delta1 power was lower than NCs, whereas beta power was higher than NCs, during four MSLT sessions. Theta and sigma power existed significant group × session interaction and simple session effects. In NCs, theta power in session 1 and session 3 were higher than in session 4, and sigma power in session 1 and session 2 were higher than session 4. Sigma power in session 3 was higher than in session 4 within CIs. There were no group effects, session effects and group × session effects in delta2 and alpha power. More detailed information about Repeated-measure ANOVA is displayed in Supplementary Table 1. The results from independent *t*-tests showed that total daytime EEG power of all frequency bands were similar between NCs and CIs. Examination of group effect in daytime EEG when examined separately for wake and for sleep resulted in findings comparable to their combined analysis with significant main group effects in delta1 and beta power. See Supplementary Figure 1 for wake comparison and Supplementary Figure 2 for sleep comparison.

### Demographics and polysomnography characteristics among normal controls and chronic insomniacs subgroups defined by sleep efficiency

CIs with SE ≥ 85% and CIs with SE < 85% subgroups contained 13 (10 females) and 18 (11 females) subjects, respectively. CIs with SE ≥ 85% age from 27 to 52 years old (mean age 34.54), while CIs with SE < 85% are 22–62 years old (mean age 36.50). The Kruskal-Wallis H test or Analysis of Variance (ANOVA) was used to evaluate differences of demographic characteristics and PSG parameters among NCs and SE determined insomnia subgroups, and Bonferroni correction was used to account for the multiple two-group comparisons. Age and sex were comparable among NCs and CIs subgroups defined by 85% SE. Compared with NCs and CIs with SE ≥ 85%, CIs with SE < 85% showed longer MSL and WASO, shorter TST, NREM, N2, and REM and lower SE. However, demographics and PSG parameters were similar between NCs and CIs with SE ≥ 85%. Demographic and PSG characteristics of CIs subgroups determined by 85% SE are provided in Table 2.

**TABLE 2:** Demographics and overnight PSG measurements in normal controls and chronic insomniacs subtypes.

	Normal controls (*n* = 21)	CIs with SE ≥ 85% (*n* = 13)	CIs with SE < 85% (*n* = 18)	ν	F/χ ^2^/H	P^1^	CIs with MSL < 15 min (*n* = 15)	CIs with MSL ≥ 15 min (*n* = 16)	ν	F/χ ^2^/H	P^2^
Age (y)	34.76 (8.38)	34.54 (6.72)	36.50 (12.88)	2	0.061	0.970 ^H^	36.33 (10.93)	35.06 (10.79)	2	0.117	0.890 ^F^
Sex (m/f)	3/18	3/10	7/11	2	3.156	0.206^χ2^	5/10	5/11	2	2.793	0.247^χ2^
MSL (min)	12.20 (3.86)	13.09 (2.40)^c^	16.33 (3.45)^b^	2	7.527	0.001^F^	11.96 (1.47)^f^	17.79 (1.95)^e^	2	27.422	<0.001^H^
ESS	7.24 (4.25)	7.69 (5.25)	4.44 (4.61)	2	5.049	0.080^H^	5.67 (5.45)	5.94 (4.88)	2	1.071	0.585^H^
TST (min)	445.11 (43.77)	459.42 (35.59)^c^	355.68 (82.10)^b^	2	20.492	<0.001^H^	433.27 (59.82)	367.24 (92.08)^e^	2	10.695	0.005^H^
SE (%)	88.51 (7.86)	90.38 (3.78)^c^	69.60 (15.97)^b^	2	26.522	<0.001^H^	84.76 (9.02)	72.26 (19.00)^e^	2	11.534	0.003^H^
SOL (min)	12.52 (13.79)	11.90 (9.30)	46.31 (69.52)^b^	2	7.116	0.029^H^	10.68 (7.35)^f^	51.75 (72.26)^e^	2	10.005	0.007^H^
WASO (min)	45.07 (32.75)	33.22(18.30)^c^	106.23 (56.78)^b^	2	19.067	<0.001^H^	65.51 (43.49)	85.08 (68.13)	2	3.449	0.178^H^
NREM (min)	355.01 (30.39)	363.48 (43.82)^c^	292.50 (60.16)^b^	2	12.233	<0.001^F^	347.07 (51.15)^f^	299.01 (67.37)^e^	2	6.225	0.004^F^
N1 (min)	70.26 (42.37)	58.27 (16.88)	64.59 (32.26)	2	0.173	0.917^H^	59.51 (22.41)	64.22 (30.78)	2	0.097	0.952^H^
N2 (min)	251.68 (39.62)	289.41 (48.39)^c^	195.74 (52.53)^b^	2	16.049	<0.001^F^	261.67 (65.12)^f^	210.04 (63.89)	2	3.886	0.027^F^
N3 (min)	33.10 (25.22)	15.69 (18.89)	31.89 (27.89)	2	4.356	0.113 ^H^	25.47 (25.07)	24.75 (26.69)	2	2.324	0.313^H^
REM (min)	90.25 (28.98)	95.97 (29.81)^c^	63.38 (30.89)^b^	2	5.722	0.006^F^	86.55 (24.24)	68.13 (40.04)	2	2.402	0.101^F^

PSG, polysomnography; TST, total sleep time; SOL, sleep onset latency; WASO, wake after sleep onset; N1, duration of non-rapid eye movement sleep stage 1; N2, duration of non-rapid eye movement sleep stage 2; N3, duration of non-rapid eye movement sleep stage 3; NREM, time of whole non-rapid eye movement sleep; REM, rapid eye movement sleep time; SE, sleep efficiency; MSL, mean sleep latency of four multiple sleep latency tests; ESS, Epworth Sleepiness Scale; CIs, Chronic Insomniacs. P^1^, significance among Normal Controls, CIs with SE ≥ 85% and CIs with SE < 85%; P^2^, significance among Normal Controls, CIs with MSL < 15 min and CIs with MSL ≥ 15 min. ^F^, comparison among Normal Controls and Chronic Insomnia subgroups by Analysis of Variance (ANOVA); ^H^, comparison among Normal Controls and Chronic Insomnia subgroups by Kruskal-Wallis H test; ^χ2^, comparison among Normal Controls and Chronic Insomnia subgroups by Chi-square (χ^2^) test; ^b^statistically significant in post hoc analysis with Bonferroni correction between Normal Controls and CIs with SE < 85%; ^c^statistically significant in post hoc analysis with Bonferroni correction between CIs with SE ≥ 85% and CIs with SE < 85%; ^e^statistically significant in post hoc analysis with Bonferroni correction between Normal Controls and CIs with MSL > 15 min; ^f^statistically significant in post hoc analysis with Bonferroni correction between CIs with MSL < 15 min and MSL ≥ 15 min.

### Beta power among normal controls and chronic insomniacs subgroups defined by sleep efficiency

**FIGURE 3:**
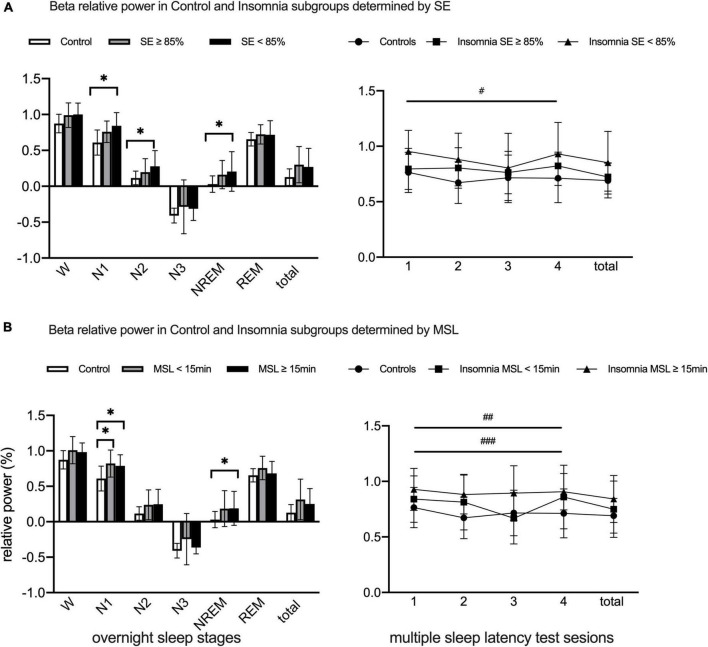
Lg10-transformed beta relative power during overnight PSG and MSLT in Normal Controls and Chronic Insomniacs subgroups. **(A)** Present beta relative power differences during overnight and daytime between Normal Controls and Insomniacs with SE ≥ 85% and SE < 85%. **(B)** Show beta relative power differences during overnight and daytime between Normal Controls and Insomniacs with MSL < 15 min and MSL > 15 min. SE, sleep efficiency; MSL, mean sleep latency; W, 2 min before sleep onset; N1, non-rapid eye movement sleep stage 1; N2, non-rapid eye movement sleep stage 2; N3, non-rapid eye movement sleep stage 3; NREM, total non-rapid eye movement sleep including N1, N2 and N3; REM, rapid eye movement sleep; Total of overnight, including W and all sleep stages in overnight. Sessions, consecutive four multiple sleep latency tests. Total, the sum of four multiple sleep latency test in relative power. beta, 16.0–30.0 Hz. *Statistically significant in group differences during overnight PSG; ^#^Statistically significant between normal controls and insomniacs with SE < 85% in *post hoc* analysis of main group effect; ^##^Statistically significant between normal controls and insomniacs with MSL ≥ 15 min in *post hoc* analysis of main group effect; ^###^Statistically significant between insomniacs with MSL < 15 min and insomniacs with MSL ≥ 15 min in *post hoc* analysis of main group effect.

Figure 3A provided group differences in beta power during overnight (evaluated by ANOVA) and presented effects of group, session, and group × session interaction in beta power during MSLT (evaluated by repeated-measures ANOVA with *post hoc* analysis of within-group and between-group effects). When compared with NCs, beta power significantly elevated in CIs with SE < 85% group during N1, N2, and NREM stages. Absence of session and group × session interaction effects, significant main group effect existed in beta power during MSLT sessions; CIs with SE < 85% presented higher beta power relative to NCs.

Comparison of power in other frequency bands among NCs, CIs with SE ≥ 85% and CIs with SE < 85% during overnight PSG and MSLT were presented in Supplementary Figures 3–7. During MSLT sessions, delta 1 and delta 2 presented main group effect, and CIs with SE < 85% showed lower delta 1 power than NCs in *post hoc* analysis. Theta and sigma power showed significant group × session effect and simple session effect. Group differences among NCs and SE defined CIs subgroups showed no significant statistical differences in other frequency bands during overnight sleep stages and MSLT sessions.

### Demographics and polysomnography characteristics among normal controls and chronic insomniacs subgroups defined by mean sleep latency

CIs with MSL < 15 min included 15 participants (10 females) between 24 and 61 years old (mean age 36.33) and CIs with MSL ≥ 15 min contained 16 subjects (11 females) between 22 and 62 years old (mean age 35.06). The Kruskal-Wallis H test or Analysis of Variance (ANOVA) was used to evaluate differences of demographic characteristics and PSG parameters among NCs and MSL determined insomnia subgroups, and Bonferroni correction was used to account for every multiple two-group comparisons. Age and sex were comparable among NCs and MSL defined CIs subgroups. CIs with MSL ≥ 15 min participants had longer MSL and SOL, shorter TST and NREM, lower SE, when compared to NCs. CIs with MSL ≥ 15 min subjects showed longer MSL and SOL, shorter NREM and N2 than CIs with MSL < 15 min. NCs and CIs with MSL < 15 min had similar demographics and overnight PSG characteristics. Specific demographics and overnight PSG measurements in NCs and MSL defined CIs subgroups are displayed in Table 2.

### Beta power among normal controls and chronic insomniacs subgroups defined by mean sleep latency

Beta power in N1 obviously increased in both CIs with MSL < 15 min and CIs with MSL ≥ 15 min groups, compared with NCs, while MSL ≥ 15 min CIs showed higher beta power in NREM sleep stages (evaluated by ANOVA). During MSLT sessions, main group effect existed in beta power (evaluated by repeated-measures ANOVA), and, in *post hoc* analysis, beta power in CIs with MSL ≥ 15 min was higher than in NCs and CIs with MSL < 15 min (Figure 3B).

Specific group differences in other frequency bands could be found in Supplementary Figures 3–7. The results showed that delta2 power increased in CIs with MSL ≥ 15 min during N1 compared to NCs, while delta1, theta, alpha and sigma power were comparable among NCs, CIs with MSL < 15 min and CIs with MSL ≥ 15 min. During MSLT sessions, delta 1 and delta 2 showed significant main group effect, and delta 2 power elevated in CIs with MSL ≥ 15 min relative to NCs and CIs with MSL < 15 min.

### General linear regression analysis between beta power and polysomnography parameters

In all subjects, total daytime beta was significantly and positively associated with total overnight beta and MSL, and marginally negatively related to SE. In all subjects, total overnight beta power was significantly and negatively associated SE. In CIs, a significant positive correlation was observed between nighttime beta and daytime beta (Table 3).

**TABLE 3:** Results from general linear regression analysis on beta relative power and SE and MSL during total overnight and daytime.

Dependent variables	Independent variables	NCs			CIs			All		
		*F*	β	*P*	*F*	β	*P*	*F*	β	*P*
Total daytime beta	Total overnight beta	0.894	0.212	0.356	8.851	0.484	0.006*	14.653	0.476	<0.001*
	SE	1.601	0.279	0.221	2.772	–0.295	0.107	3.662	–0.261	0.061^#^
	MSL	0.277	0.120	0.605	2.076	0.258	0.160	4.044	0.274	0.050*
Total overnight beta	SE	0.111	–0.076	0.743	1.372	–0.213	0.251	4.552	–0.289	0.038*
	MSL	0.010	–0.022	0.923	0.205	–0.084	0.654	0.209	0.065	0.649

β, standard regression coefficient; NCs, Normal Controls; P, value for significance of each regression coefficient; CIs, Chronic Insomniacs; MSLT, multiple sleep latency tests; SE, sleep efficiency; MSL, mean sleep latency; beta, 16.0–30.0 Hz. Values in bold indicate statistically significant; *P ≤ 0.05; ^#^, 0.05 < *P* ≤ 0.1.

## Discussion

To our knowledge, this is the first study to quantify EEG power spectral across both overnight sleep and MSLT of the following day in the same insomnia and controls subjects. The results demonstrate that patients with insomnia exhibited increased beta power during overnight sleep and the next daytime, which demonstrates that 24-h cortical hyperarousal is present in insomnia patients. Furthermore, this study found that daytime cortical hyperarousal is prominent in insomnia patients with objective low SE and long MSL.

The spectral analysis was performed on overnight EEG, significant higher beta power in CIs during W, N1, N2, NREM, and whole night, which were consistent with data from previous studies (Merica, 1998; Perlis et al., 2001a; Corsi-Cabrera et al., 2012; Spiegelhalder et al., 2012; Kang et al., 2018, 2022; Smagula et al., 2021; Shi et al., 2022; Xu et al., 2022; Zhang et al., 2022). In a recent meta-analysis, elevated relative beta power in W, NREM and whole night was also be demonstrated by Zhao et al; however, they did not explore EEG spectral power in specific N1, N2 and N3 stages, which differ in function and depth of sleep (Zhao et al., 2021). Our study, exploring EEG power in each sleep stage, providing more specific information regarding the PSG changes of insomnia disorder. Elevated beta power is considered as indicator of cortical hyperarousal, and beta activity seems associated with sensorimotor and cognitive process (Engel and Fries, 2010; Fernandez-Mendoza et al., 2016). Increased W, N1 and N2 beta power in CIs suggests a failure to fully terminate sensorimotor and cognitive processes when attempting to initiate sleep and during N1 and N2 sleep stages, which may be related to complaints of difficulty falling and maintaining asleep (Spiegelhalder et al., 2012). This same failure to fully terminate cognitive processes during sleep states likely also results in mistakenly judging sleep as wakefulness by CIs, potentially resulting in differences between subjective sufferings and objective measurements (Kang et al., 2018).

Group differences between NCs and CIs in other frequency bands were restricted to N1 stage, specifically, decrease delta2, increase alpha power in CIs. Lower delta activity may indicate decreased hyperpolarization of thalamocortical neurons during N1 sleep and less sleep quality (Lamarche and Ogilvie, 1997; Knyazev, 2012; Long et al., 2021). Synchronization in alpha frequency range plays an important role in the top-down control of cortical activation and reflects inhibition, which inhibition helps neurons in distributed networks to effectively activate common target cells and helps to established a highly selective activation pattern (Klimesch et al., 2007). High power as inhibition during wakefulness, elevated alpha power of chronic insomnias in N1 can be deemed as activation during falling asleep, which may suggest impairment of the regular falling asleep process in chronic insomniacs (Riedner et al., 2016; Rezaei et al., 2019).

In this paper we explored discriminations of MSLT EEG between NCs and CIs. CIs showed longer MSL, which is consistent with the previous papers (Bonnet and Arand, 2000; Huang et al., 2012). MSLT is commonly used to assess the propensity of falling asleep in daytime and is a valid measure of physiological arousal (Li et al., 2015). Lower MSL suggests higher sleep drive and lower physiological arousal level, whereas higher MSL indicates lower sleep drive and higher physiological arousal level (Bonnet and Arand, 2005). Meanwhile, as an indicator of cortical hyperarousal, beta power showed main group effect and has been significantly increased in MSLT session relative to NCs. Together with extended MSL and enhanced nighttime and daytime beta power, the findings of current study demonstrate that chronic insomnia patients present both daytime physiological and cortical hyperarousal, supporting the notion of 24-h cortical hyperarousal.

Delta activity, as a measure of sleep intensity, is associated positively with arousal threshold and subjective sleep quality, and negatively with indices of physiological arousal (Borbely, 1982; Neckelmann and Ursin, 1993; Kurylenko et al., 2015; Long et al., 2021). Delta2 activity is generated thalamocortical neurons and delta1 is generated cortically, independent of the thalamus (Steriade et al., 1993; Steriade, 2006; Knyazev, 2012). Furthermore, EEG activity in delta1 rather than delta2 band is more functionally relevant for cognitive function (Staresina et al., 2015). In our study, delta 1 power was lower in CIs during MSLT sessions, when compared with NCs, which indicate that CIs showed less sleep intensity and higher physiological arousal level in daytime. Meanwhile, when examined separately for wake and for sleep in MSLT resulted in comparable findings to their combined analysis, which indicated that the findings of daytime EEG spectral analysis were robust and daytime hyperarousal was present for both the awake and sleeping state in chronic insomniacs.

When exploring the 24-h cortical hyperarousal level in various insomnia subtypes, we identified an elevated overnight cortical hyperarousal level in all insomnia subtypes but more prominent in insomniacs with lower SE and longer MSL, which is consistent with previous studies (Spiegelhalder et al., 2012; Fernandez-Mendoza et al., 2016). On the contrary, increased daytime cortical hyperarousal level presented in insomniacs with worse SE and longer MSL. This is new in the literature and may indicate that daytime cortical hyperarousal is more prominent in insomnia subtypes of shorter objective sleep duration (measured by lower SE) and greater physiological hyperarousal. Elevated cortical arousal may co-exist with physical arousal during daytime.

Results from general linear regression analysis showed that significantly positive correlation between nighttime beta and daytime beta power has been found in all subjects and CIs, which suggests that subjects with higher nighttime beta have higher daytime beta, indicating cortical hyperarousal is a round-the-clock phenomenon. Significantly negative correlation between nighttime beta and SE and marginally negative correlation between daytime beta and SE have been found in all subjects, which supports that insomniacs with objective short sleep duration appear to exhibit higher nighttime and daytime cortical arousal. Meanwhile, elevated daytime cortical arousal may co-exist with physical arousal, as evidenced by significantly positive correlation between MSL and daytime beta power in all subjects. However, the expected correlations between beta power and SE and MSL in CIs were not observed, perhaps because of the small sample size of the current study. This should be investigated in future studies employing larger samples of insomnia patients.

The current study has limitations. First, the small number of participants in insomnia subgroups limit the possibility of generalizing the findings, our suggestive findings will need to be confirmed in larger samples. Second, previous studies suggested that males and females have different quantitative EEG sleep characteristics during sleep; for example, women with insomnia have higher beta power than man with insomnia (Buysse et al., 2008; Choi et al., 2019; Hong et al., 2021), while in healthy subjects, sex differences of EEG activity showed in delta, theta and low alpha instead of beta frequency range (Carrier et al., 2001; Mongrain et al., 2005; Feinberg et al., 2006). Thus, in our study, the imbalance of sex between groups may impact our findings, despite the lack of observed significant differences in sex between NCs and CIs. Third, given the retrospective nature of our study which analyzed EEG data of patients in Sleep Medicine Center, no adaption night was employed so potential first night effects cannot be eliminated; although previous evidence supports that quantitative EEG outcomes during sleep show high short-term stability in both good sleepers and insomniacs (Israel et al., 2012). Fourth, all patients from Sleep Medicine Center outpatient clinic primarily complained of sleep related symptoms and not of psychological or psychiatry concerns. Therefore, the physicians screened depression, anxiety or other psychological or psychiatric complaints *via* face-to-face interview without the use of standardized psychological/psychiatric examination or validated questionnaires. Although this process leaves us confident that individuals with moderate to high levels of depression or anxiety were excluded from the study, the presence of low levels of depression and anxiety in the study sample cannot be ruled out. Finally, the current study could not demonstrate whether 24-h cortical hyperarousal occurs in unison with other measures of physiological arousal, such as increased cortisol or norepinephrine or impaired heart rate variability as such measurements were not part of the clinical protocol. Thus, this possibility needs to be explored in the future studies.

## Conclusion

In conclusion, the current study demonstrated that insomnia patients showed enhanced beta activity compared to controls both during overnight sleep and throughout the following day, indicating the presence of 24-h cortical hyperarousal. A further unique finding was that daytime cortical hyperarousal was most prominently exhibited in patients with objective short sleep duration or great physical hyperarousal. Our results support the conception of 24-h cortical hyperarousal in chronic insomnia, adding to our understanding of neurophysiology of insomnia and suggesting the simultaneous suppression of both night and daytime arousal level may become a new target for the treatment of insomnia.

## Data availability statement

The original contributions presented in this study are included in the article/Supplementary material, further inquiries can be directed to the corresponding author/s.

## Ethics statement

The studies involving human participants were reviewed and approved by the Research Ethics Board of the West China Hospital of Sichuan University. The patients/participants provided their written informed consent to participate in this study.

## Author contributions

YS, RR, and XT: conception and design of study. YS, RR, YZ, and FL: acquisition of data. YS, MV, and XT: analysis and/or interpretation of data. MV and XT: revising the manuscript critically for important intellectual content. YS, RR, FL, YZ, MV, and XT: approval of the version of the manuscript to be published. All authors contributed to the article and approved the submitted version.

## Footnotes

1
https://www.synwing.com/
