# Supplementary material for: Elevated beta activity in the nighttime sleep and multiple sleep latency electroencephalograms of chronic insomnia patients
Source: Front Neurosci. 2022 Nov 2;16:1045934. doi: 10.3389/fnins.2022.1045934 (PMC9667071; doi:10.3389/fnins.2022.1045934)
Supplement: Supplementary file 1 [file Data_Sheet_1.docx]

Supplementary Material


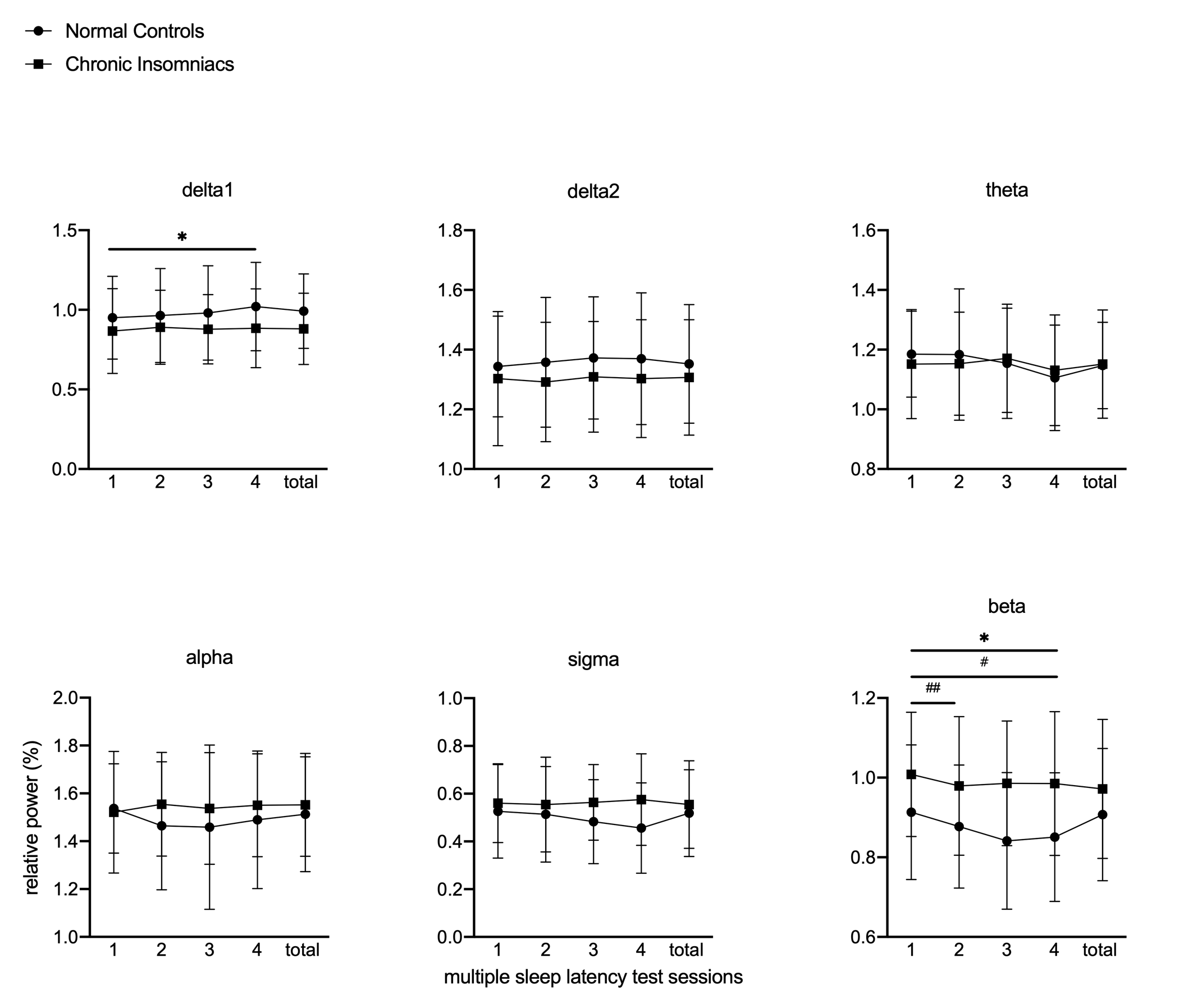


**Supplementary Figure 1.** Lg10-transformed relative power of all frequency bands during MSLT wake stage in Normal Controls and Chronic Insomniacs. Wake stage, two minutes before sleep onset. Sessions, consecutive four multiple sleep latency tests. Total, the sum of four multiple sleep latency tests in relative power. delta1, 0.5-1.0Hz; delta2, 1.0-4.0Hz; theta, 4.0-8.0Hz; alpha, 8.0-13.0Hz; sigma, 13.0Hz-16.0Hz; beta, 16.0-30.0Hz. ^*^, statistically significant in main group effect; ^#^, statistically significant in main session effect; ^##^, statistically significant between session 1 and session 2 in post hoc analysis of main session effect.


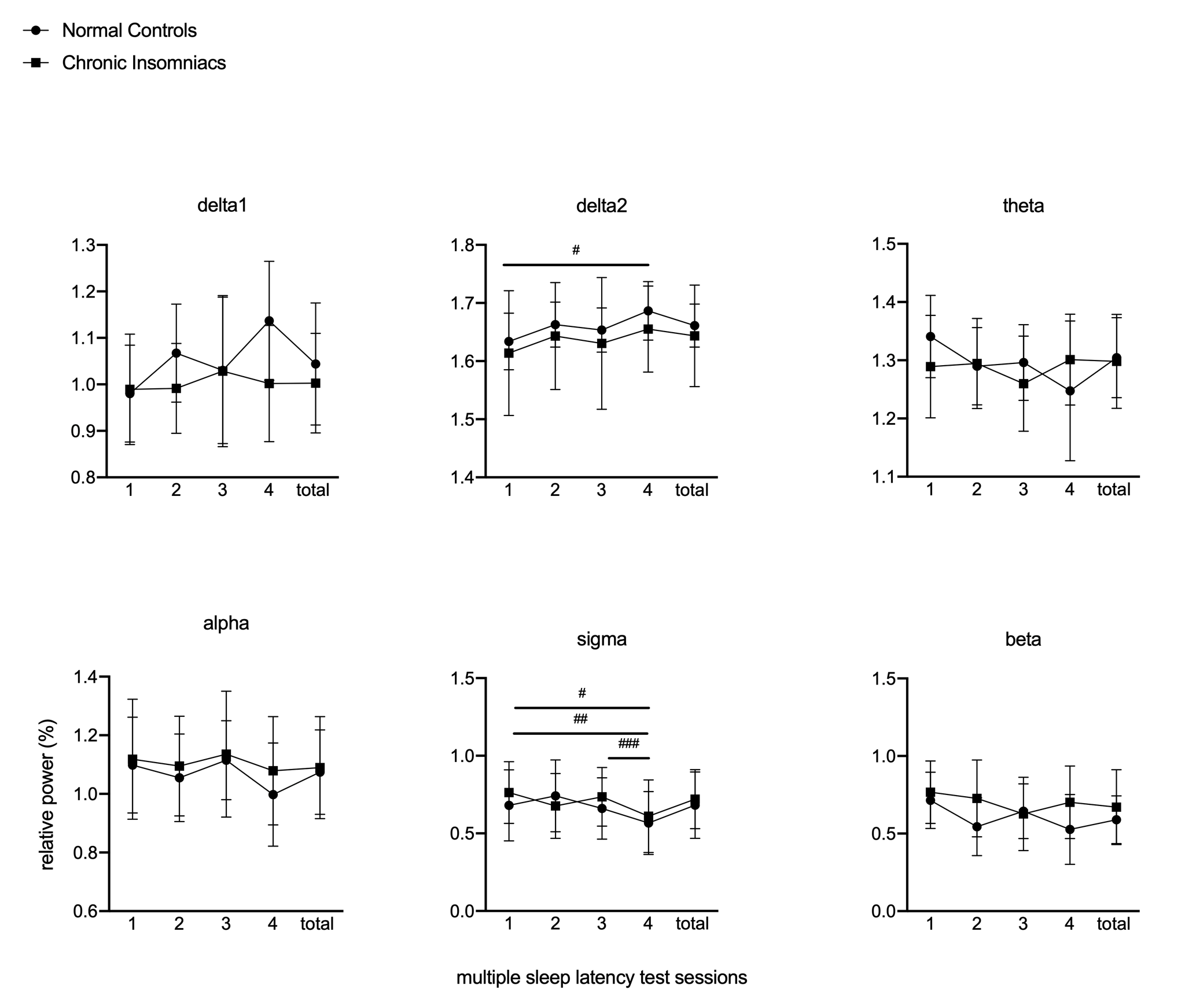


**Supplementary Figure 2.** Lg10-transformed relative power of all frequency bands during MSLT sleep stages in Normal Controls and Chronic Insomniacs. Sleep stages, containing all sleep stages during one MSLT session. Sessions, consecutive four multiple sleep latency tests. Total, the sum of four multiple sleep latency tests in relative power. delta1, 0.4-1.0Hz; delta2, 1.0-4.0Hz; theta, 4.0-8.0Hz; alpha, 8.0-13.0Hz; sigma, 13.0Hz-16.0Hz; beta, 16.0-30.0Hz. ^#^, statistically significant in main session effect; ^##^, statistically significant between session 1 and session 4 in post hoc analysis of main session effect; ^###^, statistically significant between session 3 and session 4 in post hoc analysis of main session effect.


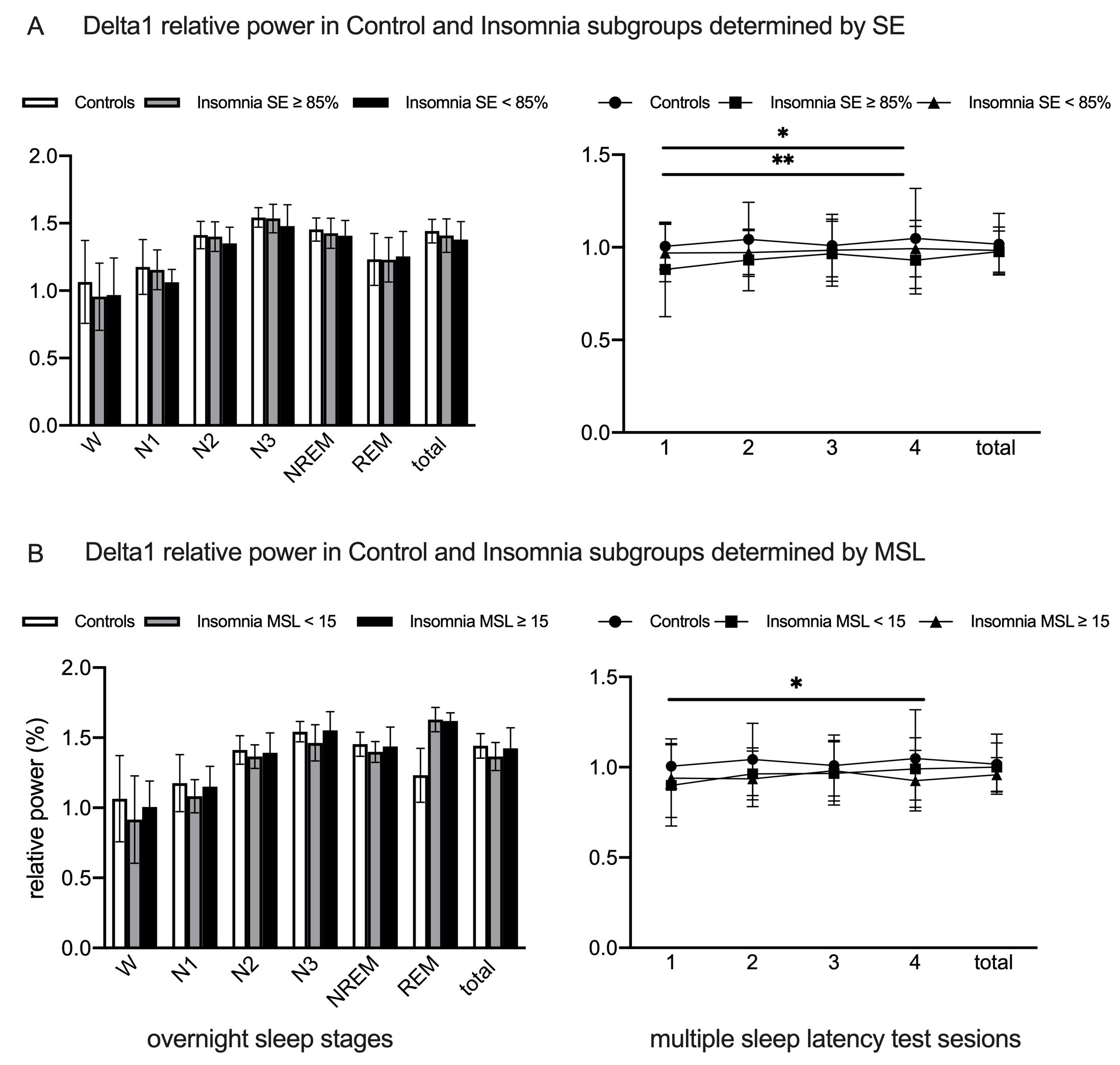


**Supplementary Figure 3.** Lg10-transformed delta1 relative power during overnight PSG and MSLT in Normal Controls and Chronic Insomniacs subgroups. Sessions, consecutive four multiple sleep latency tests. Total, the sum of four multiple sleep latency tests in relative power. delta1, 0.4-1.0Hz. ^*^, statistically significant in main group effect; ^**^, statistically significant between normal controls and insomniacs with SE < 85% in post hoc analysis of main group effect.


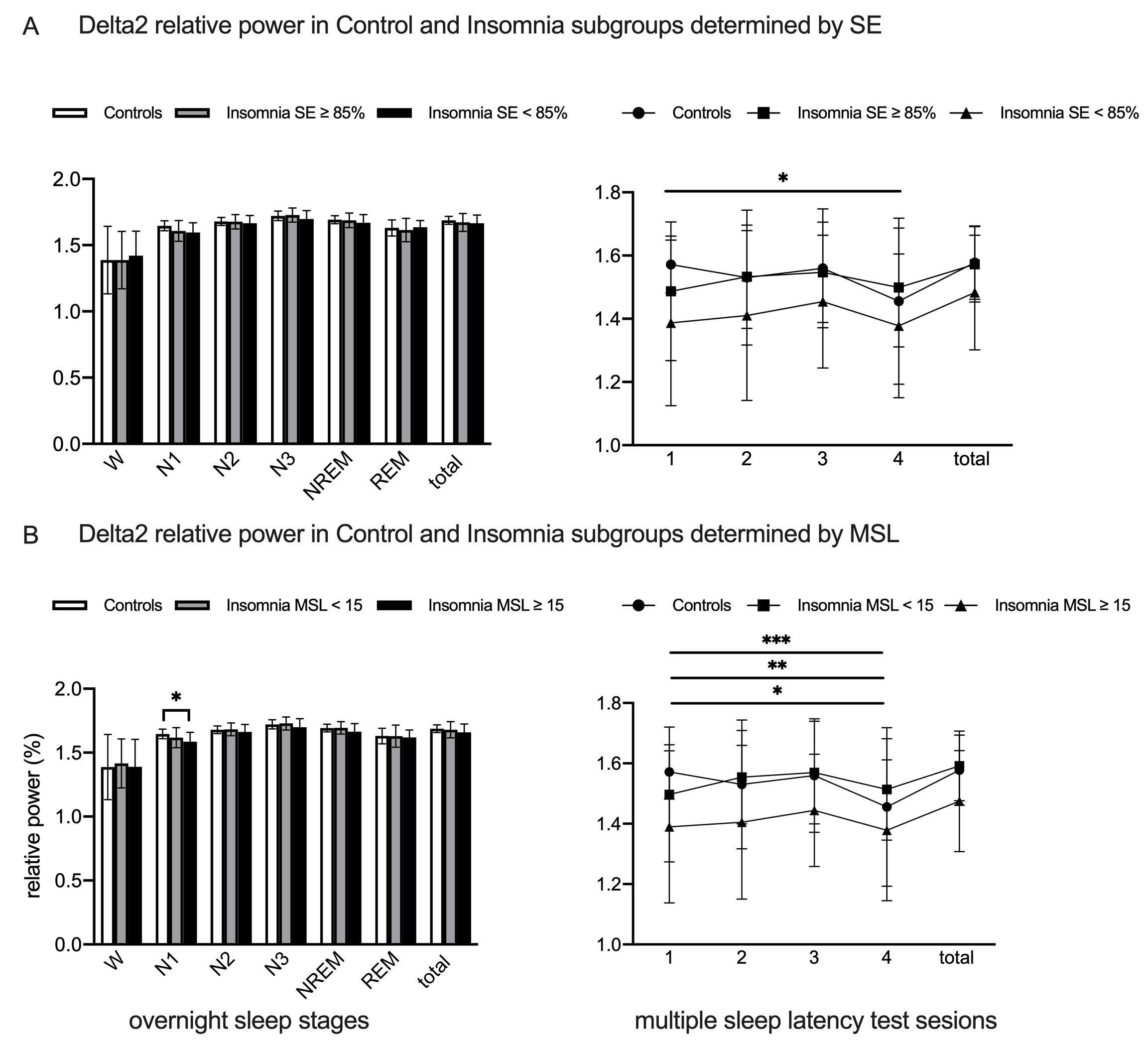


**Supplementary Figure 4.** Lg10-transformed delta2 relative power during overnight PSG and MSLT in Normal Controls and Chronic Insomniacs subgroups. Sessions, consecutive four multiple sleep latency tests. Total, the sum of four multiple sleep latency tests in relative power. delta2, 1.0-4.0Hz. ^*^, statistically significant in main group effect; ^**^, statistically significant between normal controls and insomniacs with MSL ≥ 15min in post hoc analysis of main group effect; ^***^, statistically significant between insomniac with MSL < 15min and insomniacs with MSL ≥ 15min in post hoc analysis of main group effect.


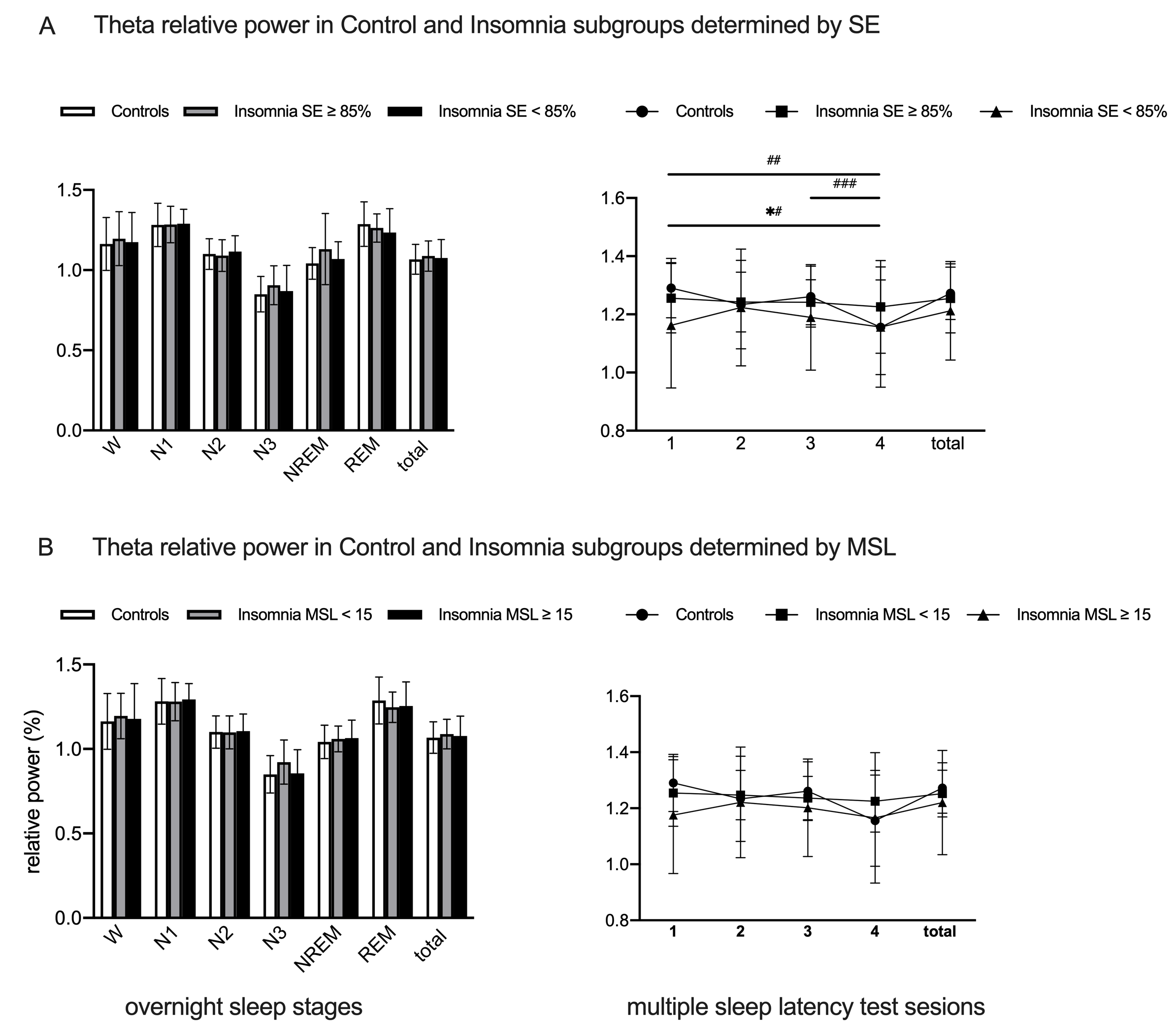


**Supplementary Figure 5.** Lg10-transformed theta relative power during overnight PSG and MSLT in Normal Controls and Chronic Insomniacs subgroups. Sessions, consecutive four multiple sleep latency tests. Total, the sum of four multiple sleep latency tests in relative power. theta, 4.0-8.0Hz. ^*#^, statistically significant in group x session effect; ^##^, statistically significant between session 1 and session 4 in post hoc analysis of simple group effect within normal controls; ^###^, statistically significant between session 3 and session 4 in post hoc analysis of simple group effect within normal controls.


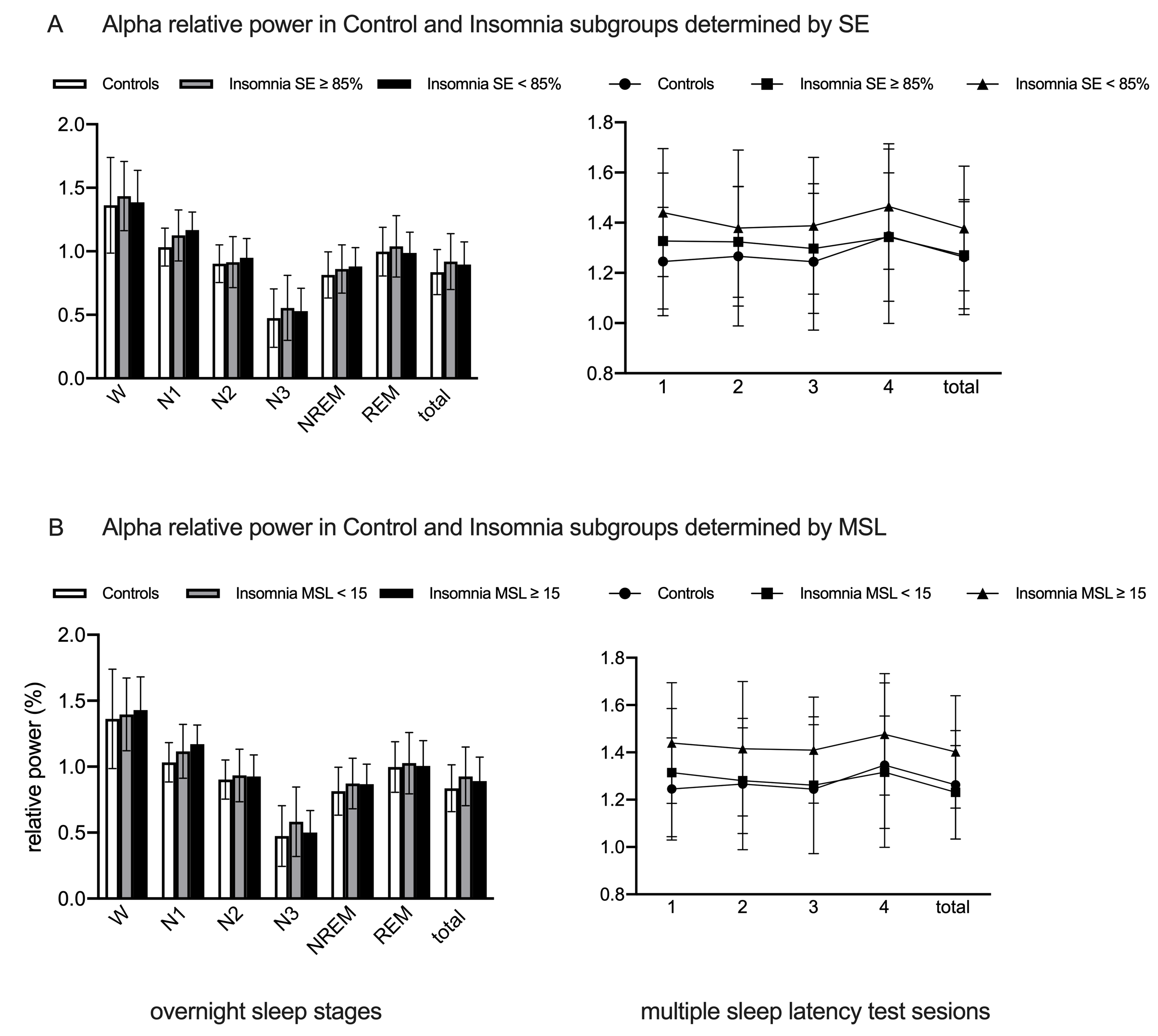


**Supplementary Figure 6.** Lg10-transformed alpha relative power during overnight PSG and MSLT in Normal Controls and Chronic Insomniacs subgroups. Sessions, consecutive four multiple sleep latency tests. Total, the sum of four multiple sleep latency tests in relative power. alpha, 8.0-13.0Hz.


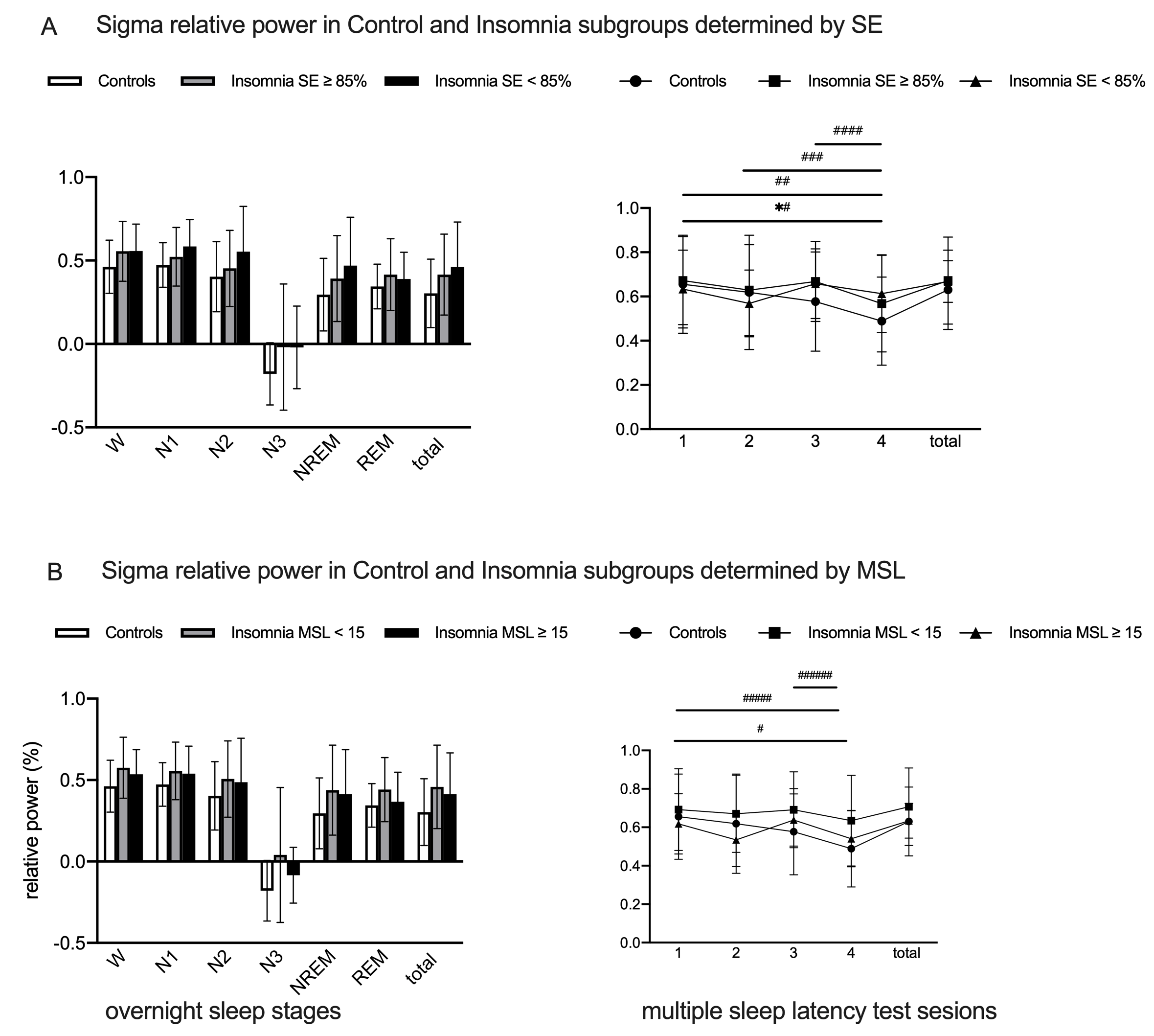


**Supplementary Figure 7.** Lg10-transformed sigma relative power during overnight PSG and MSLT in Normal Controls and Chronic Insomniacs subgroups. Sessions, consecutive four multiple sleep latency tests. Total, the sum of four multiple sleep latency tests in relative power. sigma, 13.0Hz-16.0Hz. ^*#^, statistically significant in group x session effect; ^##^, statistically significant between session 1 and session 4 in post hoc analysis of simple group effect within normal controls; ^###^, statistically significant between session 2 and session 4 in post hoc analysis of simple group effect within normal controls; ^####^, statistically significant between session 3 and session 4 in post hoc analysis of simple group effect within insomniacs with SE ≥ 85%; ^#^, statistically significant in main session effect; ^#####^, statistically significant between session 1 and session 4 in post hoc analysis of main session effect; ^######^, statistically significant between session 3 and session 4 in post hoc analysis of main session effect.

| Supplementary Table1. Relative power of all frequency bands during MSLT in Normal Controls and Chronic Insomniacs | | | | | | | | | | | | | | | | | | |
| --- | --- | --- | --- | --- | --- | --- | --- | --- | --- | --- | --- | --- | --- | --- | --- | --- | --- | --- |
|  |  | Normal Controls  (n=21) |  |  | Chronic Insomniacs  (n=31) |  |  | Session effect | | |  | Group effect | | |  | Session x group effect | | |
|  |  | Mean (SD) | P |  | Mean (SD) | P |  | ν | F | P |  | ν | F/t | P |  | ν | F | P |
| delta1 |  |  |  |  |  |  |  | 3 | 2.476 | 0.064 |  | 1 | 6.596 | 0.013 |  | 3 | 1.957 | 0.123 |
| session1 |  | 10.42(3.11) |  |  | 9.23(3.61) |  |  |  |  |  |  |  |  |  |  |  |  |  |
| session2 |  | 11.62(4.57) |  |  | 9.41(3.14) |  |  |  |  |  |  |  |  |  |  |  |  |  |
| session3 |  | 10.98(4.49) |  |  | 10.09(3.62) |  |  |  |  |  |  |  |  |  |  |  |  |  |
| session4 |  | 13.53(6.48) |  |  | 9.90(3.89) |  |  |  |  |  |  |  |  |  |  |  |  |  |
|  |  |  |  |  |  |  |  |  |  |  |  |  |  |  |  |  |  |  |
| delta2 |  |  |  |  |  |  |  | 3 | 1.291 | 0.281 |  | 1 | 2.535 | 0.118 |  | 3 | 0.352 | 0.750 |
| session1 |  | 38.04(7.21) |  |  | 31.29(13.15) |  |  |  |  |  |  |  |  |  |  |  |  |  |
| session2 |  | 37.09(12.84) |  |  | 33.48(13.25) |  |  |  |  |  |  |  |  |  |  |  |  |  |
| session3 |  | 38.86(11.70) |  |  | 34.69(13.08) |  |  |  |  |  |  |  |  |  |  |  |  |  |
| session4 |  | 33.18(16.20) |  |  | 30.99(13.29) |  |  |  |  |  |  |  |  |  |  |  |  |  |
|  |  |  |  |  |  |  |  |  |  |  |  |  |  |  |  |  |  |  |
| theta |  |  | 0.578a |  |  | 1.000a |  | 3 | 4.003 | 0.009 |  | 1 | 0.053 | 0.818 |  | 3 | 2.884 | 0.038 |
| session1 |  | 20.00(4.36) | 1.000b |  | 17.66(6.81) | 1.000b |  |  |  |  |  | 1 | 1.536 | 0.221 |  |  |  |  |
| session2 |  | 18.08(5.68) | 0.001c |  | 18.18(6.47) | 1.000c |  |  |  |  |  | 1 | 0.048 | 0.827 |  |  |  |  |
| session3 |  | 18.71(4.13) | 1.000d |  | 17.32(5.46) | 1.000d |  |  |  |  |  | 1 | 0.809 | 0.373 |  |  |  |  |
| session4 |  | 15.27(5.59) | 0.194e |  | 17.05(7.22) | 1.000e |  |  |  |  |  | 1 | 0.775 | 0.383 |  |  |  |  |
|  |  |  | 0.042f |  |  | 1.000f |  |  |  |  |  |  |  |  |  |  |  |  |
| alpha |  |  |  |  |  |  |  | 3 | 1.208 | 0.309 |  | 1 | 2.370 | 0.130 |  | 3 | 0.257 | 0.857 |
| session1 |  | 19.82(10.03) |  |  | 28.40(16.83) |  |  |  |  |  |  |  |  |  |  |  |  |  |
| session2 |  | 22.70(16.59) |  |  | 26.57(16.58) |  |  |  |  |  |  |  |  |  |  |  |  |  |
| session3 |  | 21.32(14.93) |  |  | 25.84(14.94) |  |  |  |  |  |  |  |  |  |  |  |  |  |
| session4 |  | 29.30(20.71) |  |  | 29.15(16.15) |  |  |  |  |  |  |  |  |  |  |  |  |  |
|  |  |  |  |  |  |  |  |  |  |  |  |  |  |  |  |  |  |  |
| sigma |  |  | 1.000a |  |  | 0.332a |  | 3 | 6.245 | 0.001 |  | 1 | 0.339 | 0.563 |  | 3 | 2.924 | 0.036 |
| session1 |  | 5.20(3.20) | 0.367b |  | 4.94(2.14) | 1.000b |  |  |  |  |  | 1 | 0.343 | 0.776 |  |  |  |  |
| session2 |  | 4.97(3.27) | 0.006c |  | 4.39(2.11) | 0.383c |  |  |  |  |  | 1 | 1.689 | 0.370 |  |  |  |  |
| session3 |  | 4.29(2.31) | 1.000d |  | 4.95(1.94) | 0.240d |  |  |  |  |  | 1 | 1.427 | 0.103 |  |  |  |  |
| session4 |  | 3.44(1.91) | 0.024e |  | 4.30(2.13) | 1.000e |  |  |  |  |  | 1 | 1.347 | 0.158 |  |  |  |  |
|  |  |  | 0.299f |  |  | 0.046f |  |  |  |  |  |  |  |  |  |  |  |  |
| beta |  |  |  |  |  |  |  | 3 | 1.645 | 0.190 |  | 1 | 8.412 | 0.006 |  | 3 | 0.705 | 0.551 |
| session1 |  | 6.40(3.54) |  |  | 8.56(4.51) |  |  |  |  |  |  |  |  |  |  |  |  |  |
| session2 |  | 5.10(2.03) |  |  | 8.00(4.69) |  |  |  |  |  |  |  |  |  |  |  |  |  |
| session3 |  | 5.84(3.63) |  |  | 7.11(3.79) |  |  |  |  |  |  |  |  |  |  |  |  |  |
| session4 |  | 5.74(2.56) |  |  | 8.73(4.86) |  |  |  |  |  |  |  |  |  |  |  |  |  |

Repeated measurement analysis of variance was used to assess effects of group, session and group * session interaction. a, comparison between session1 and session2; b, comparison between session1 and session2; c, comparison between session1 and session2; d, comparison between session2 and session3; e, comparison between session2 and session4; f, comparison session3 and session4. MSLT, multiple sleep latency tests; SD, standard deviation. delta1, 0.5-1.0Hz; delta2, 1.0-4.0Hz; theta, 4.0-8.0Hz; alpha, 8.0-13.0Hz; sigma, 13.0Hz-16.0Hz; beta, 16.0-30.0Hz.
